# Inclusion of tumor periphery in radiomics analysis of magnetic resonance images does not improve predictions of preoperative therapy response in patients with rectal cancer

**DOI:** 10.1007/s00261-025-04815-0

**Published:** 2025-02-05

**Authors:** Nafsika Korsavidou Hult, Sambit Tarai, Klara Hammarström, Joel Kullberg, Elin Lundström, Tomas Bjerner, Bengt Glimelius, Håkan Ahlström

**Affiliations:** 1 Radiology, Department of Surgical Sciences, Uppsala University, Akademiska Sjukhuset, Ingång 70, Uppsala, 751 85 Sweden; 2 Department of Immunology, Genetics and Pathology, Uppsala University, Dag Hammarskjölds v 20, Uppsala, 751 85 Sweden; 3 Dept. of Health, Medicine and Caring Sciences (HMV), Division of Diagnostics and Specialist Medicine (DISP), Linköping University, Linköping, 581 83 Sweden; 4 Antaros Medical AB, GoCo House Entreprenörsstråket 10, Mölndal, 431 53 Sweden

**Keywords:** Radiomics, Rectal cancer, pCR, NAR, Recurrence, MRI

## Abstract

**Background/Purpose:**

To evaluate the advantages of including versus excluding the tumor periphery and combining diffusion-weighted imaging (DWI) with T2-weighted imaging (T2w) for outcome predictions of preoperative radio(chemo)therapy in rectal cancer.

**Methods:**

Four analysis strategies, based on two segmentation methods and two magnetic resonance imaging (MRI) sequences, were evaluated in 106 patients examined with pretreatment MRI. One segmentation method included the tumor periphery in the region of interest (ROI) encompassing the whole tumor (wROI), considered as the reference segmentation approach, and one included only the central part (cROI). Relevant radiomics imaging features were extracted from either T2w alone or from both T2w and DWI and used by a machine learning algorithm for the prediction of pathologic complete response (pCR), neoadjuvant rectal (NAR) score, and disease recurrence. The area under the curve (AUC) was the performance measure. AUCs were compared with a bootstrapping method based on 10^4^ bootstraps.

**Results:**

cROI applied to both T2w and DWI provided the highest numerical prediction of pCR (AUC 0.76), however, not significantly superior to the other strategies (*p* ≥ 0.138). cROI applied to both T2w and DWI also yielded the highest numerical prediction of NAR score (AUC 0.84), showing advantages over wROI-based analysis strategies (AUC 0.66 and 0.69; *p* ≤ 0.008). When compared to cROI applied to T2w alone (AUC 0.73), the benefit was borderline statistically significant (p = *0.053*). For prediction of disease recurrence, no differences were found between the analysis strategies.

**Conclusions:**

Inclusion of the tumor periphery in radiomic analysis of magnetic resonance images does not improve predictions of the preoperative therapy response in patients with rectal cancer. Excluding tumor periphery while adding DWI to T2w improves prediction of the NAR score, although it does not affect pCR or recurrence prediction.

**Graphical abstract:**

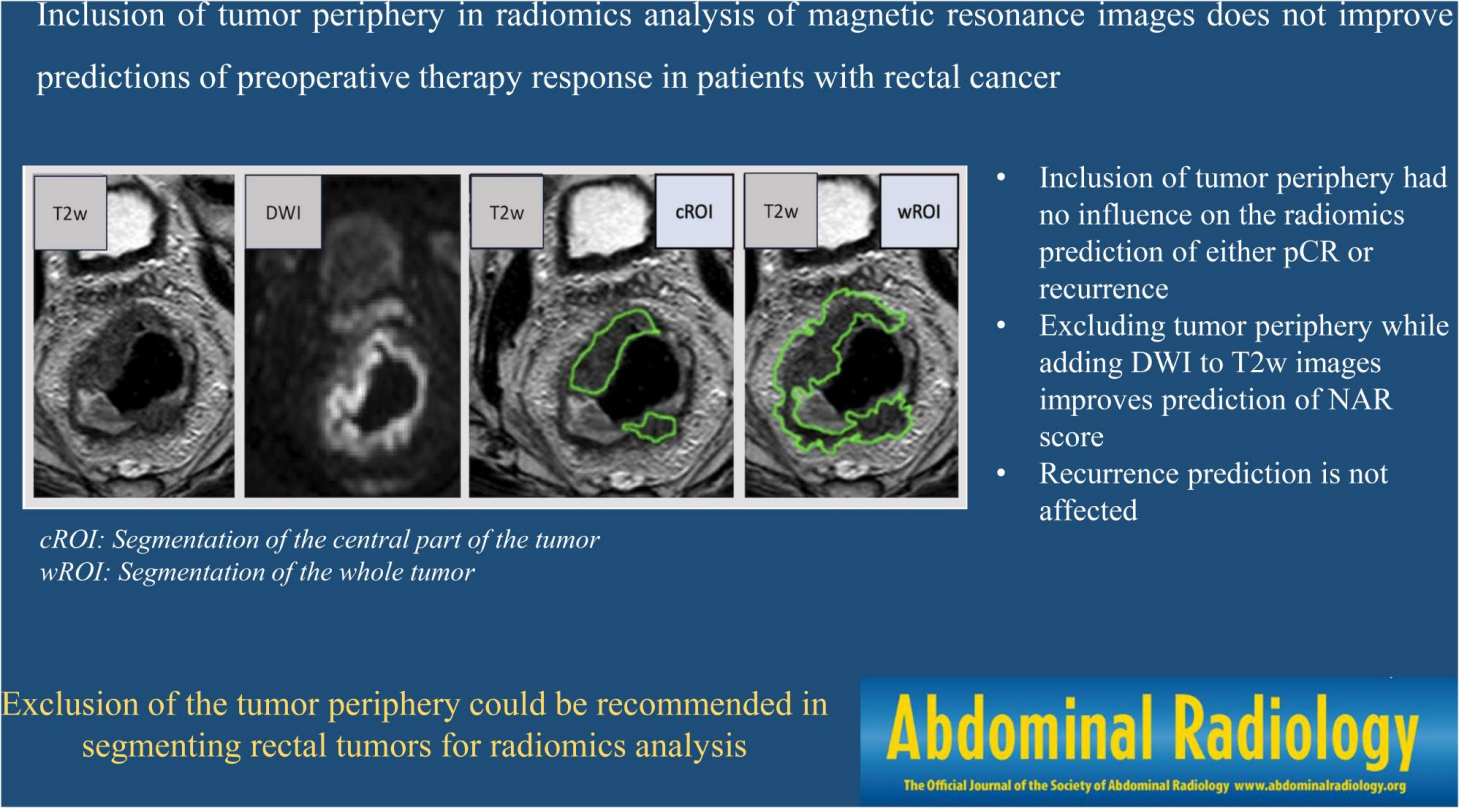

**Supplementary Information:**

The online version contains supplementary material available at 10.1007/s00261-025-04815-0.

## Background

Intratumor heterogeneity in colorectal cancer (CRC) refers to the variation in internal tumor architecture between distinctive regions of the same tumor [1]. Separate tumor regions contain cells with dissimilar characteristics and locally identifiable subclones with different properties including separate mutations [2,3]. The more heterogenous the clusters are, the higher the malignancy grade and the risk of metastasis or recurrence after treatment [4]. CRC growth patterns have been identified as expansile, intermediate and infiltrative. They correlate with survival, with infiltrative growth patterns being associated with worse rates [3]. This suggests that the outermost infiltrative part of the tumor may contain valuable prognostic information. Moreover, the presence of lymphocytic infiltration is associated with higher survival rates [5,6]. The macroscopic growth patterns have been correlated with prognosis in stage III CRC patients [7]. Many rectal cancers (RCs), constituting approximately one-third of all CRC, are upfront treated with radiotherapy (RT) or chemoradiotherapy (CRT) to lower the recurrence rates, improve survival and, most recently, to defer surgery in cases of excellent treatment response (complete clinical response, cCR). If operated on, some of these patients have no remaining tumor cells in the specimen (pathological complete response, pCR). PCR, or cCR, i.e. no signs of remaining tumor either after palpation, endoscopically or on magnetic resonance imaging (MRI) are both signs of an excellent response to the treatment provided [8] and allow the patient to enter a watch and wait (W&W) program for organ preservation [9]. Both pCR and cCR are also associated with a low risk of recurrence either locoregionally or at distant sites. The neoadjuvant rectal score (NAR) score [10] has emerged as another early response parameter integrating both the end-result, pathologic tumor T and nodal N stage (ypTN) and the starting point, clinical tumor (cT) stage in the evaluation of long-term outcomes after different treatments [11,12] used also in this study. Thus, both pCR and NAR score are relevant endpoints in the exploration of markers associated with response. Long-term outcomes, such as recurrence, disease-free survival and overall survival, are clinically of even greater importance but also influenced by many other factors than immediate treatment response.

MRI is currently the most valuable staging tool for selecting patients with RC for different treatments [13,14]. The widely used computational research method of radiomics is a tool for the assessment of quantitative tumor features from medical images, which extends beyond the manual assessment by radiologists. In radiomics, machine learning strategies extract tumor texture features, using the spatial distribution of voxel intensities from predefined segmentations, in large imaging datasets [15]. In RC, radiomics has shown promise in the prediction of both pCR [16,17,18,19] and the NAR score [17,20].

Most radiomics studies on RC assume that all parts of the tumor should be included in the segmentation. Some studies have also investigated whether information from the mesorectal space with tumor extensions, lymph nodes and extramural venous invasion could increase the capacity in predicting outcomes [7,18,21,22]. In a breast cancer study, exploring different parts of the tumors, peritumoral radiomic features better discriminated human epidermal growth factor receptor 2 (HER2) expression, in comparison to intratumoral features [23]. The value of including versus excluding the tumor periphery in radiomics analysis of RC has not yet been investigated thoroughly. Most commonly, radiomics feature extraction is conducted on T2-weighted MR images (T2w), rich in anatomical details. The potential value of combined T2w and diffusion-weighted imaging (DWI), the latter containing information on tissue microstructure obtained from the diffusion patterns of water molecules, has been explored [18,24].

The aim of this study was to evaluate the potential advantages of including versus excluding the tumor periphery and combining DWI with T2w for radiomics outcome prediction of preoperative/neoadjuvant therapy (RT/CRT) in locally advanced RC.

## Materials and methods

### Study population

This retrospective study was approved by the regional ethical research committee. Patients diagnosed with RC between 2010 and 2018 were included after informed written consent. Inclusion criteria were primary, non-metastasized RC, staged with MRI and treated with either short-course RT, conventional CRT or a total neoadjuvant treatment (TNT), followed by delayed abdominal surgery. Multiparametric MR images of the rectum of 369 patients with histologically diagnosed rectal adenocarcinoma considered locally advanced requiring pretreatment were evaluated [25]. The images were acquired at three different institutions, using standardized MRI protocols. Due to local variations in T2w and/or DWI acquisitions or due to intravenous contrast agent, 264 patients were excluded. One additional patient was excluded at a later stage due to a benign classification of the lesion at histopathology. After exclusions, 106 patients remained. They were treated with either (1)short-course RT (scRT) to 5 × 5 Gy in one week with a delay of 6–8 weeks prior to surgery, (2)CRT to approximately 50 Gy for 5 weeks with concomitant fluoropyrimidine, frequently capecitabine, or (3)total neoadjuvant treatment (TNT) using scRT followed by 4–6 cycles of CAPOX (capecitabine and oxaliplatin) [26,27]. The clinical TN stage was based on the pretreatment MRI. The pathological stage was assessed by histologic analysis after surgical excision and any incidence of recurrence (local or distant) was extracted from the patients’ medical files and the Swedish colorectal cancer registry.

### MRI of rectum and tumor segmentation

According to recommendations from the European Society of Gastrointestinal Imaging, MR rectum examinations should be standardized with respect to the acquisition parameters [13]. The images accepted for study inclusion are axial T2w with 3 mm slice thickness and axial DWI with 5 mm slice thickness and including b value = 800 s/mm^2^, acquired on 1.5 and 3.0 Tesla systems from different vendors.

**Fig. 1:**
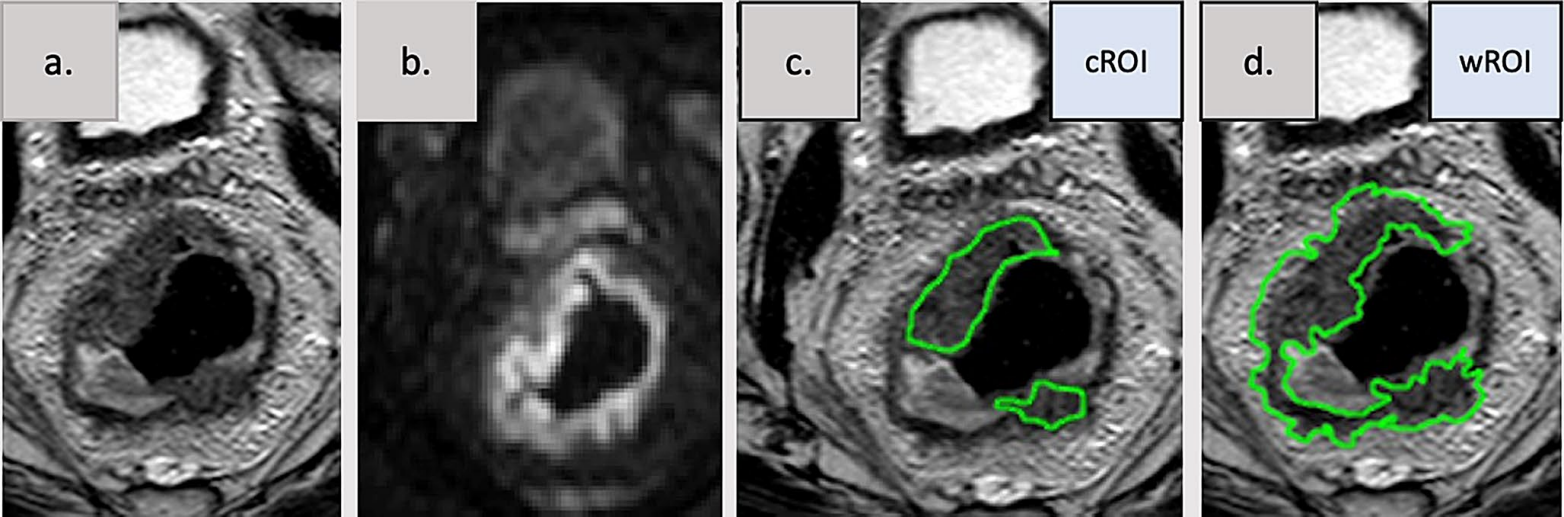
Example of rectal tumor segmentations. Segmentations on axial T2-weighted images (T2w) with a freehand region of interest (ROI). The central (cROI) segmentation includes the tumor bulk excluding the outermost margin and the small extension in the mesorectal compartment, whereas the whole (wROI) segmentation includes tumor bulk and the periphery of the tumor as well as its extensions in the mesorectal compartment. **a**. T2-weighted images, **b**. diffusion weighted image (DWI) at the same anatomical position as the T2w, **c**. T2-weighted images with ROI of central part of tumor (cROI) with segmentation considering the diffusion information, **d**. T2-weighted images (wROI) including the tumor periphery

Tumors were segmented manually, with free-hand regions of interest (ROIs) in the pretreatment T2w, by a board-certified radiologist with 7 years of experience in RC (NKH). The segmentations were performed in every slice where the tumor was visible. The reader was blinded to the clinical and pathological TNM stages and all outcomes. The segmentations were performed by two approaches, as listed below, with care taken to avoid inclusion of air and tumor necrosis, example shown in Fig. 1.

#### Segmentation of the whole tumor, wROI

The tumor was first delineated to its outmost margin in the T2w images, including also the tumor extensions into the mesorectum. Very thin mesorectal extensions, however, were excluded to avoid fat-containing voxels.

#### Segmentation of the central part of the tumor, cROI

The tumor was then delineated in the T2w images also using DWI by only including tumor voxels with restricted diffusion (i.e. high signal in the DWI, spatially registered to the T2w) and by excluding the 2–4 mm tumor rim (i.e. tumor periphery) according to the T2w. To obtain a cROI that was distinct from wROI, the thickness of the defined tumor periphery depended on the bulk size of the tumor; larger bulks allowed for thicker (~ 4 mm) peripheries whereas smaller bulks required thinner (~ 2 mm) peripheries.

Further image examples of tumor segmentations are available in the Supplementary material 2.

## Outcomes

### Pathologic complete response (pCR)

If no viable tumor cells were observed in the surgical specimen (ypT0N0), the tumor was classified as showing pCR. All other cases were considered as non-pCR, thus creating a binary classification.

### Neoadjuvant rectal (NAR) score

The NAR score (NAR = [5pN-3cT-pT + 12]^2^/9.61), was based on the cT status before treatment, and the ypT combined with ypN after neoadjuvant CRT/RT. In our study, the NAR scores ranged between 0 and 65. Typically, the NAR score values are binned into 3 categories: low (< 8), intermediate (8–16), and high (> 16). To perform a binary analysis, owing to the limited number of patients and imbalance in the number of patients between the groups, the intermediate and low groups were merged and hereafter referred to as the low/intermediate group.

### Disease recurrence

Any recurrence, locoregional or distant, was considered. Patients were routinely followed after 1 and 3 years clinically, with computed tomography (CT) of the thorax and the abdomen, and with carcinoembryonic antigen (CEA) blood tests according to the national guidelines [28]. Colonoscopy was performed after 5 years.

### Radiomics

**Fig. 2:**
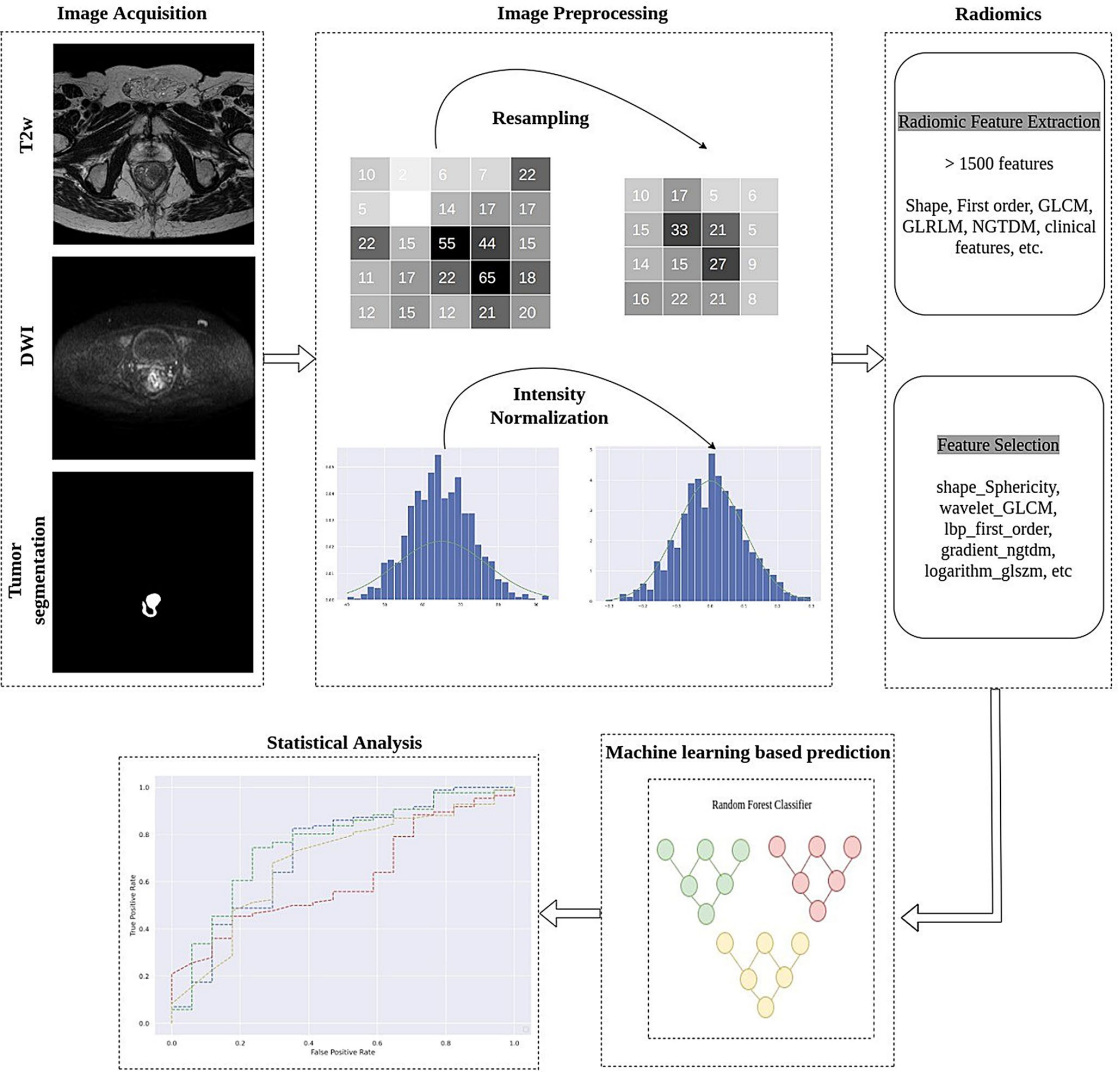
Schematic overview of an end-to-end framework for radiomic analysis

The radiomic method consisted of a number of processing steps, which form an end-to-end framework using the tumor segmentations and MR images as input data for prediction of the outcome parameters. An overview of the framework is presented in Fig. 2.

### Image preprocessing

Image preprocessing is a crucial step before extracting radiomic features, to compensate for varying voxel intensities due to differences in image acquisition parameters and MR systems. To address variability across imaging systems, histogram matching was applied along with normalization and resampling. All T2w and DWI images were resampled to the same voxel size (the same spacing) to standardize the images. The target voxel size [0.5, 0.5, 3]mm^3^, to which all images were resampled, was chosen based on the most commonly occurring voxel size. Finally, image intensities were normalized between 0 and 1 for all patients.

### Radiomic feature extraction

Radiomic tumor features were extracted from the preprocessed T2w and DWI, within the two segmentations (wROI and cROI), using the open-source Python package PyRadiomics library (version 3.0.1). Features were extracted separately for each pair of segmentation and image. Prior to feature extraction, 8 different filters were applied to the original images (i.e., the T2w and DWI), including Laplacian of Gaussian (LoG), exponential, logarithmic, gradient, wavelet, square, square root and local binary pattern (LBP) filters. This resulted in a total of 9 different image types, for each of T2w and DWI, including the original images. From each image type, the radiomic features were extracted, except shape features, which were only extracted from the original images. Totally, 1578 radiomic features were extracted from each image-segmentation pair. Feature-specific normalization was applied independently to ensure that all radiomic features laid within a comparable range of values Supplements material 1.

### Feature selection, classification and machine learning-based prediction

Three state-of-the-art methods for the selection of relevant features for outcome prediction (pCR, NAR, recurrence) were compared: (1)minimum redundancy maximum relevance (mRMR), (2)least absolute shrinkage and selection operator (LASSO) and (3)logistic regression. Feature extraction and selection were applied independently during the prediction of pCR, NAR and recurrence. The main purpose of this step was to remove redundant features not providing additional information and select only the top independent features. Thereafter, machine learning models, such as random forest and logistic regression (with L1-regularization), were trained on the selected features for the prediction of outcomes. In total, six different combinations of feature selection algorithms and machine learning classifiers were compared (Table 1) and the combination with the highest precision was chosen for further analysis [29].

**Table 1:** Machine learning models for feature selection and machine learning classifiers. The highest accuracy, sensitivity and precision values are marked in bold

Feature Selection	Machine Learning Classifier	Accuracy	Precision	Sensitivity
***pCR***				
mRMR	Random Forest	0.6893	0.6736	0.7185
mRMR	Logistic Regression	0.7134	0.7069	0.7345
LASSO	Random Forest	0.8252	0.7590	0.8013
***LASSO***	***Logistic Regression***	***0.8544***	***0.8395***	0.8258
Logistic Regression	Random Forest	0.8349	0.7125	0.8231
Logistic Regression	Logistic Regression	0.8217	0.7889	***0.8345***
***NAR score***				
mRMR	Random Forest	0.7043	0.6138	0.7338
mRMR	Logistic Regression	0.7058	0.6235	0.7043
LASSO	Random Forest	0.7247	0.6315	0.7622
LASSO	Logistic Regression	0.7629	0.7132	0.7723
Logistic Regression	Random Forest	0.7339	0.7023	0.7435
***Logistic Regression***	***Logistic Regression***	***0.8381***	***0.8336***	***0.8428***
***Recurrence***				
mRMR	Random Forest	0.7732	0.6842	0.7585
mRMR	Logistic Regression	0.7650	0.7053	0.7763
LASSO	Random Forest	0.8185	0.7678	0.7835
***LASSO***	***Logistic Regression***	0.8525	***0.7985***	0.7648
Logistic Regression	Random Forest	***0.8583***	0.7741	***0.8018***
Logistic Regression	Logistic Regression	0.8462	0.7840	0.7946

pCR: pathological complete response, NAR: neoadjuvant rectal score, mRMR: minimum redundancy maximum relevance, LASSO: Least absolute shrinkage and selector operator

Feature selection and machine learning-based outcome prediction were applied independently during the prediction of pCR, NAR score, and recurrence. A machine learning classifier was used to categorize each subject according to pCR vs. non-pCR, low/intermediate vs. high NAR score and recurrence vs. no recurrence, based on the features derived from the following combinations of segmentations and MR images shown in Table 2.

**Table 2:** Segmentation alternatives, denomination and the included images in each segmentation alternative

Segmentation	Segmentation name	Included images
wROI	wROI + T2w	T2w
wROI	wROI + T2w + DWI	T2w and DWI
cROI	cROI + T2w	T2w
cROI	cROI + T2w + DWI	T2w and DWI

wROI: whole tumor segmentation, cROI: central tumor segmentation, ROI: Region of interest, T2w: T2 weighted, DWI: diffusion weighted images

The machine learning model was evaluated using 4-fold cross-validation, stratified based on the predicted output, which maintained the same class imbalance ratio among folds as in the original dataset.

### Statistics

For all predictions, receiver-operating characteristic (ROC) curve analyses were performed and the area under the curve (AUC) was calculated. If not specified otherwise, the results are presented as AUC (95% confidence interval, 95% CI) with the 95% CIs calculated from 10^4^ bootstrap samples of the individual patients’ predictions. The differences in AUC among the four methods (segmentation + image combination) were evaluated by bootstrapping based on the same 10^4^ bootstrap samples. Fischer’s exact test was performed to investigate potential differences in recurrence between the pCR and non-pCR groups and between the low/intermediate and high NAR groups. Statistical tests were two-sided and *p* values < 0.05 were considered statistically significant. No correction for multiple comparisons was conducted. Analyses were performed in R version 4.2.3 using the pROC package.

## Results

Table 3 presents patient characteristics, distribution of patients between outcome groups and treatments. For the 71 patients who were alive at the end-study date, the median follow-up time was 8.1 years (range 5–10 years).

**Table 3:** Patient characteristics, clinical stage, pathological stage and treatment alternatives

	All	Response groups		NAR score groups			Recurrence groups	
		pCR	Non-pCR	Low	Intermediate	High	Rec	Non-Rec
n	106	17 (16%)*	89 (84%)*	22 (21%)*	49 (46%)*	35 (33%)*	29 (27%)*	77 (73%)*
Age	66 ± 11	64 ± 11	67 ± 11	63 ± 11	66 ± 11	69 ± 11	67 ± 10	66 ± 11
Males	64 (60%)*	9 (14%)	55 (86%)	12 (19%)	32 (50%)	20 (31%)	18 (28%)	46 (72%)
**Clinical stage**								
cT1 and cT2	6 (6%)*	1 (17%)	5 (83%)	1 (17%)	3 (50%)	2 (33%)	2 (33%)	4 (67%)
cT3 and cT4	100 (94%)*	16 (16%)	84 (84%)	21 (21%)	46 (46%)	33 (33%)	27 (27%)	73 (73%)
cN0	13 (12%)*	2 (15%)	11 (85%)	2 (15%)	9 (69%)	2 (15%)	2 (15%)	11 (85%)
cN 1–2	93 (88%)*	15 (16%)	78 (84%)	20 (22%)	40 (43%)	33 (35%)	27 (29%)	66 (71%)
**Preoperative treatment**								
scRT	35 (33%)*	3 (9%)	32 (91%)	3 (9%)	18(51%)	14 (40%)	9 (26%)	26 (74%)
CRT	47 (44%)*	8 (17%)	39 (83%)	13 (28%)	19(40%)	15 (32%)	12 (26%)	35 (74%)
TNT	24 (23%)*	6 (25%)	18 (75%)	6 (25%)	12 (50%)	6 (25%)	8 (33%)	16 (67%)
**Pathological stage**								
ypT0	19 (18%)*	17 (89%)	2 (11%)	18 (95%)	0 (0%)	1 (5%)	2 (11%)	17 (89%)
ypT1 and ypT2	29 (27%)*	0 (0%)	29 (100%)	4 (14%)	22 (76%)	3 (10%)	6 (11%)	23 (79%)
ypT3 and ypT4	58 (55%)*	0 (0%)	58 (100%)	0 (0%)	27 (47%)	31 (53%)	21 (36%)	37 (64%)
ypN0	68 (64%)*	17 (25%)	51 (75%)	21 (31%)	45 (66%)	2 (3%)	13 (19%)	55 (81%)
ypN1-2	38 (36%)*	0 (0%)	38(100%)	1 (3%)	4 (11%)	33 (87%)	16 (42%)	22 (58%)
**Postoperative treatment**								
	39 (37%)*	4 (24%)	35 (39%)	7 (32%)	16 (33%)	16 (46%)	11 (38%)	28 (36%)

*Percentages calculated relative to the total number of patients (*n* = 106). Remaining percentages calculated relative to the total number of patients within each group of therapy response, NAR score and recurrence. pCR: pathological complete response, NAR score: neoadjuvant rectal score, c: clinical stage, y: pathological stage after treatment, scRT: short course radiotherapy, CRT: chemoradiotherapy, TNT: total neoadjuvant therapy, age in [mean ± SD]

### Prediction of pathologic complete response

**Fig. 3:**
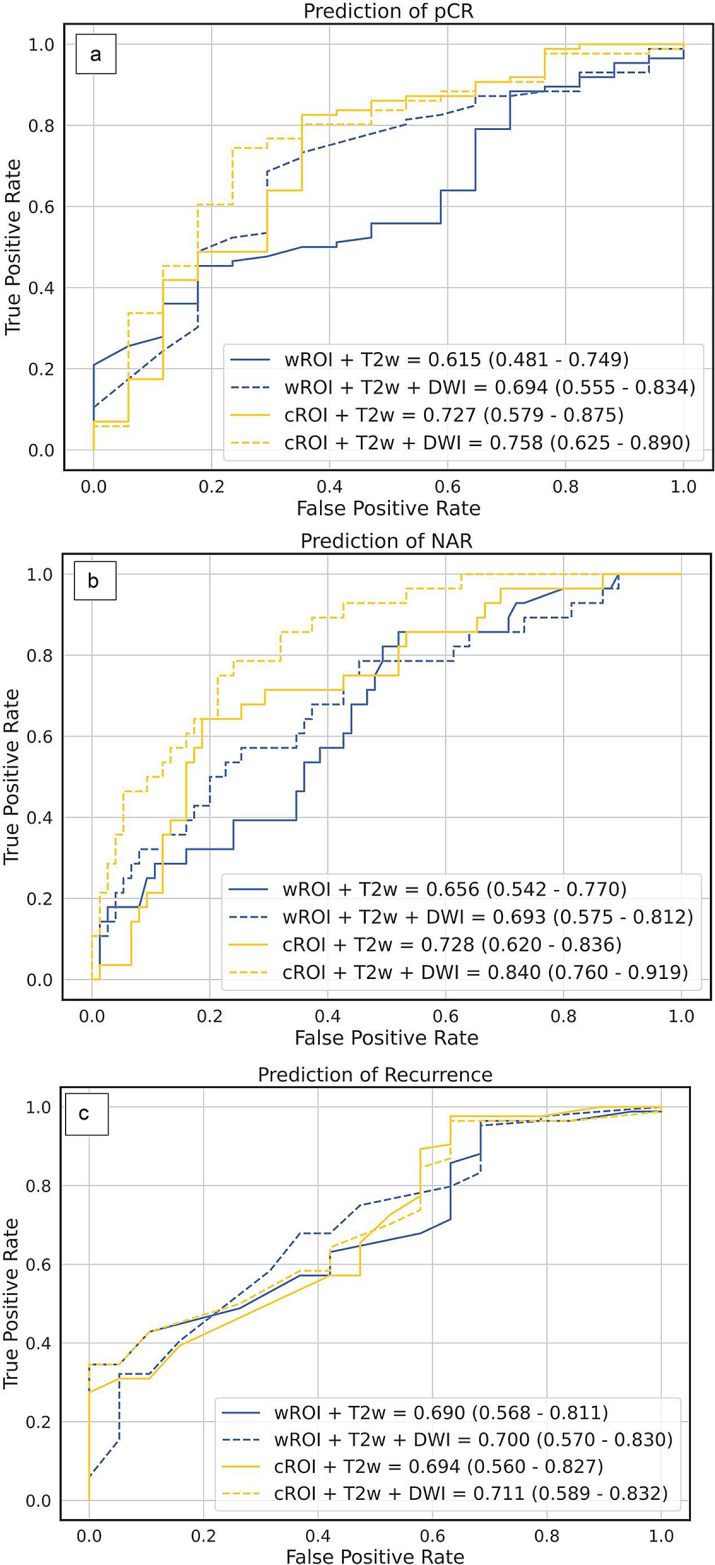
The calculated areas under the curves (AUCs) for the prediction of outcomes **a**. pathological complete response (pCR), **b**. neoadjuvant rectal (NAR) score and **c**. disease recurrence, based on different combinations of segmentation and MR images AUCs and 95% confidence intervals (CIs) in parentheses

**Fig. 4:**
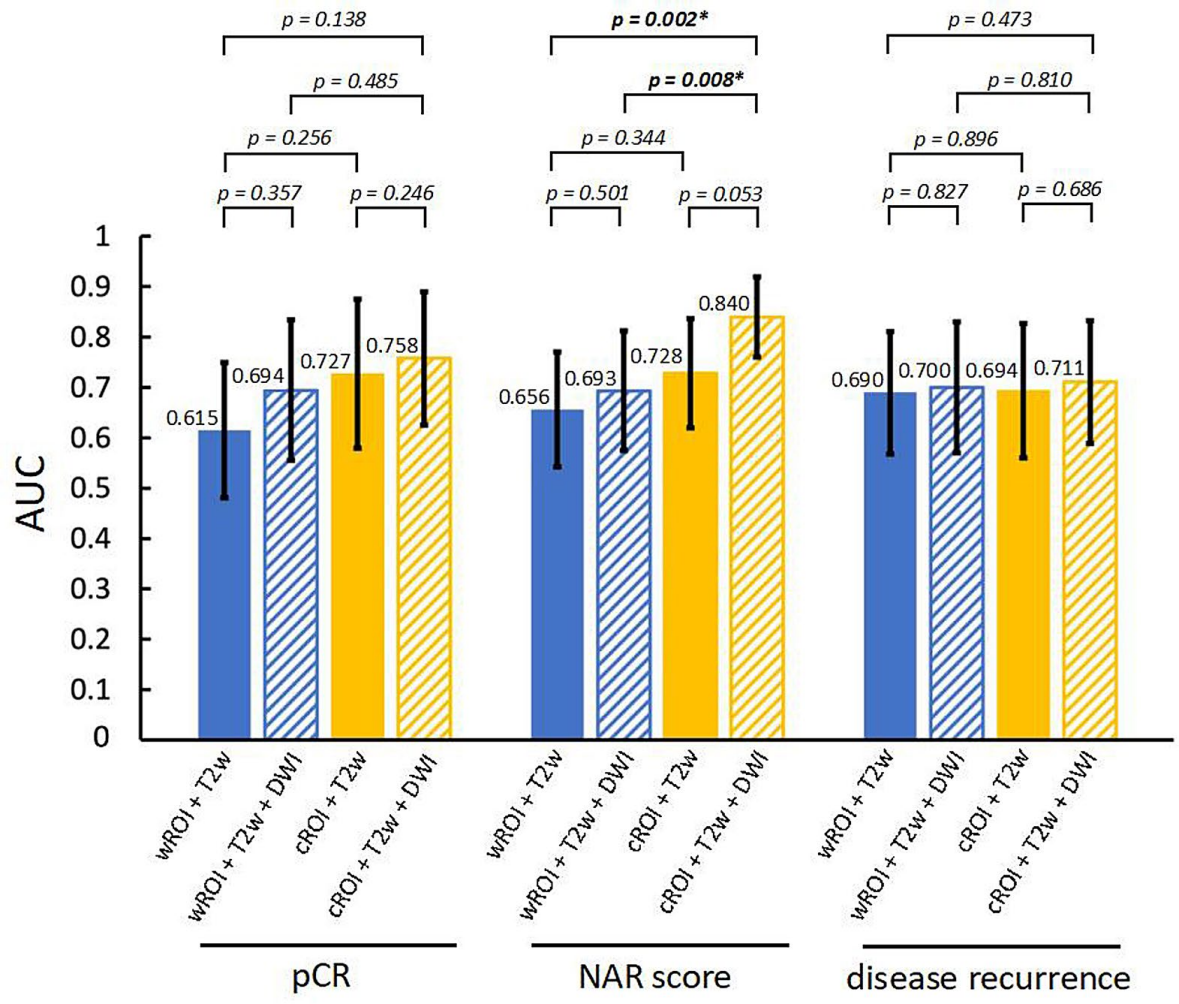
Comparisons between area under the curve (AUC) values for outcome prediction of pathologic complete response (pCR), the neoadjuvant rectal (NAR) score and disease recurrence, which are based on different combinations of segmentations (wROI and cROI) and MR images (T2w + DWI)

Among the 106 patients, 17 (16%) achieved pCR. The feature selection method LASSO together with logistic regression as a machine learning classifier presented the highest precision (0.84), Table 1. For this combination, the accuracy was 0.85 and the sensitivity was 0.82. This algorithm returned 20 features for prediction of pCR. AUC values for the different combinations of segmentation and images, obtained from Lasso/logistic regression, are presented in Fig. 3a. The highest AUC (0.76) was achieved with the segmentation-image combination (cROI + T2w + DWI) and the lowest AUC (0.62) with the reference method wROI + T2w, Fig. 3a. None of the AUCs from the segmentation-image combinations was statistically significantly different from the rest (*p*>0.138), Fig. 4.

### Prediction of the NAR score

Among the 106 patients, 22 (21%) were categorized into the low NAR score group, 49 (46%) into the intermediate NAR score group and 35 (33%) into the high NAR score group. The logistic regression as a feature selection choice and machine learning classifier presented the highest precision (0.83), Table 1. For this combination, the accuracy was 0.85 and the sensitivity was 0.84. The feature selection algorithm returned 24 features for NAR prediction. The highest AUC (0.84) was obtained with cROI + T2w + DWI and the lowest AUC (0.66) with the reference method wROI + T2w, presented in Fig. 3b. Furthermore, the combined approach of cROI + T2w + DWI showed statistically significant advantages over the other approaches, although the benefit with respect to cROI + T2w was only borderline (*p* = 0.053), Fig. 4.

### Prediction of recurrence

Among the 106 patients, 29 (27%) experienced recurrence. No significant difference in recurrence was observed between patients in the non-pCR group (27/89 (30%)) and those in the pCR group (2/17 (12%)), (*p* = 0.154). Recurrence was more common in the NAR high group (16/35 patients (46%)) than in the NAR low/intermediate group (13/71 patients (18%)), (*p* = 0.005). The feature selection method LASSO together with logistic regression as a machine learning classifier, presented the highest precision (0.79), Table 1. For this combination, the accuracy was 0.85 and the sensitivity was 0.76. The feature selection algorithm returned 20 features. The highest AUC (0.71) was obtained with cROI + T2w + DWI and the lowest AUC (0.69) with the reference method wROI + T2w, Fig. 3c. None of the compared pairs were significantly different from the others, as shown in Fig. 4.

Error bars represent 95% confidence intervals (95% CIs). Values on bars represent the calculated AUC values. Statistical significance (*p* < 0.05). CIs values shown in Fig. 3 for each outcome.

## Discussion

Inclusion versus exclusion of the tumor periphery in the tumor ROI did not significantly affect the radiomics-based prediction of the efficacy marker pCR after preoperative treatment in locally advanced RC. Neither was prediction of pCR influenced by the chosen MR image combination. However, exclusion of the tumor periphery combined with T2w and DWI showed advantages for prediction of the NAR score, a complementary surrogate marker of preoperative treatment efficacy. The prediction of long-term outcomes, represented by disease recurrence, was not significantly affected by either the segmentation method or MRI approach.

Our anticipation was that marked heterogeneity within the tumor rim, containing a mixture of inflammatory cells, fibrotic tissue, and mostly rather few tumor cells, could provide important information for the pCR prediction. On the contrary, this infiltration zone showed no added value in the prediction of response to treatment, but rather diluted the information provided by the bulk of the tumor. Partial volume effects from adjacent tissue, resulting from limitations in the manual delineation combined with a finite spatial resolution, may contribute to the decrease in the predictive capacity. Such partial volume effects might be more severe for more advanced and/or high-grade tumors due to their frequently diffuse infiltrative peripheral borders. The addition of DWI to the reference T2w MRI approach, combined with exclusion of the tumor periphery, slightly improved the prediction of NAR score. This is possibly due to the high sensitivity of DWI to differences in tissue microstructure, and thereby the potential ability to reflect heterogeneous patterns in malignant tissue and between malignant and healthy tissue. Such patterns can subsequently be captures by the radiomics model to complement patterns from the anatomical T2w. However, the lack of significant impact of DWI combined with exclusion of the tumor periphery on the prediction of pCR is not understood and warrants future investigation.

A study comparing 2D and 3D segmentation strategies for radiomics therapy outcome predictions reported comparable performances [30]. Other studies have investigated the role of tumor regions and the mesorectal compartment in radiomics-based predictions. Pizzi et. al [31] reported an AUC of 0.70 for the prediction of pCR using T2w derived radiomic features from both the tumor core (the whole tumor) and tumor border (2 mm of tumor periphery and 2 mm of the mesorectal fat). Our wROI + T2w (not including the 2 mm mesorectal fat) reached an AUC of 0.61. When the impact of only the tumor border was assessed by Pizzi, their model revealed lower classification performance with an AUC of 0.54, suggesting that inclusion of the tumor periphery and some of the mesorectal information was less important for the pCR prediction. Shaish et. al [17], reported a mean AUC of 0.80 for the prediction for pCR, derived from segmenting the entire mesorectum compared with a lower AUC when only the tumor was segmented. One interpretation of this finding is that information from the mesorectum, in terms of lymph nodes and vascular tumoral invasion as well as microtumoral clusters, could enhance the prediction of treatment response, as shown by Jayaprakasam et al [32]. Our numerically best performing approach for prediction of pCR was with the addition of DWI (cROI + T2 + DWI with an AUC 0.76), which was not included in the abovementioned studies. The study by Pizzi et al. have also added clinical parameters which increased the performance from 0.70 to 0.80. This potentially useful addition might also increase our results, but was not the scope of our study.

For NAR score, Shaish et al. reported a higher AUC from radiomics of the tumor than from the mesorectal compartment, indicating that information from the latter is less important than that from the tumor. In line with this previous study, our study showed that exclusion of the tumor periphery combined with the addition of DWI increased the performance of the model (AUC from 0.66 to 0.84). Neither exclusion of the tumor periphery alone nor the addition of DWI alone significantly increased the predictive performance compared to considering the whole tumor in T2w images only (Fig. 4).

For disease recurrence prediction, studies in the literature that investigate the influence of radiomics on different parts of the tumor are lacking. We did not find that including the tumor periphery changed the results compared with using only the tumor. However, Mc Entee et al. reported that well-established prognostic factors for disease recurrence can be present in the mesorectal compartment, as extramural venous invasion [33], whereas Karjol et al. discussed lymph node status [34]. Jayaprakasam et.al [32] showed a model performance for the prediction of local and distance recurrence (AUC 0.79 and 0.87, respectively), including segmentations solely of the mesorectum but did not evaluate potential differences between the mesorectum and the tumor itself.

There were several limitations in this study. First of all, it was a retrospective study. However, the eligible patients were consecutively retrieved from a prospective database that included patients with non-metastasized RC. The segmentations were performed manually by one radiologist, which did not allow for inter-reader comparisons. The study by Pizzi et al. has the advantage of segmenting the tumor border in an automated manner from the manual delineation of the tumor core. An automated segmentation method of both the tumor and tumor periphery (e.g., based on deep learning (DL)) could potentially have provided more standardized segmentations compared to our manual approach. Due to the limited data in our study, development of an automated segmentation method was beyond the scope of this study. The limited number of patients also restricted the possibility to use a part of the dataset as internal validation set. We nevertheless performed cross-validation with 4 folds. External validation of the prediction models, on a separate test set (e.g., from another imaging site), is warranted in the future.

The inclusion of images from different vendors is a strength, but could also introduce biases due to differences in image intensity, resolution and noise characteristics across different MR systems. These differences may cause inconsistent feature extraction, potentially leading to overfitting or poor generalization. To mitigate these biases, pre-processing techniques, including resampling and histogram matching, was used. These steps reduced MR system related differences, which facilitated for model to focus on clinically relevant features for improved generalizability. Radiomic features include complex texture descriptors, such as wavelet and lbp-derived parameters. While these high-order features may not have an immediate clinical interpretation, they likely capture subtle image patterns that could reflect underlying tumor biology be reducing reliance on manual feature selection but require significant computational resources and larger datasets, which may not be feasible with the current cohort. The applicability of DL and the interpretability of radiomic features are promising areas for future research, which could also focus on utilizing advanced feature selection algorithms, including Principal Component Analysis and Autoencoders. While traditional radiomics models could effectively extract features from tumor regions, they have limitations in capturing relationships within high-dimensional medical images. DL techniques such as convolutional neural networks can reduce reliance on manual feature selection but require significant computational resources and larger datasets, which may not be feasible with the current cohort. The applicability of DL and the interpretability of radiomic features are promising areas for future research. It will also focus on utilizing advanced feature selection algorithms, including Principal Component Analysis and Autoencoders.

A strength of our radiomics approach was the choice of feature selection algorithm and machine learning classifier, optimized for each outcome parameter with precision used as key performance metric due to the imbalanced dataset. An increased number of patients and a decreased level of image heterogeneity would likely have enhanced the statistical power and potentially also affected the statistical significance between certain comparisons, such as between the two strategies cROI + T2w and cROI + T2w + DWI for prediction of NAR, currently failing to reach significance (AUC = 0.728 and 0.840, *p* = 0.053).

To the best of our knowledge, this study is the first to evaluate the advantages of including versus excluding the tumor periphery and combining different MRI sequences for outcome predictions of preoperative treatment in RC. State-of-the-art feature selection methods were used, and internal validation was performed according to recent suggestions [35]. Methodologically, our radiomics analyses were performed to maintain generalizability and reproducibility [36,37]. Our experiments focused solely on imaging material and did not incorporate clinical parameters, with the potential to further improve the outcome predictions.

## Conclusions

Inclusion of tumor periphery in radiomic analysis of magnetic resonance images had no influence on the radiomics prediction of either pCR or recurrence after preoperative treatment in RC. As exclusion of the tumor periphery improved the prediction of the NAR score, it could therefore be recommended to not include the tumor periphery but only delineate the central part of the tumor according to our definition.

## Electronic supplementary material

Below is the link to the electronic supplementary material.

Supplementary Material 1

Supplementary Material 2

## Data Availability

The data underlying this article will be shared on reasonable request to the corresponding author.

## Abbreviations

MRI: Magnetic resonance imaging
DWI: Diffusion weighted imaging
T2w: T2 weighted imaging
ROI: Region of interest
wROI: Whole Region of interest
cROI: Central Region of interest
AUC: Area under the curve
W&W: Watch and wait
ypTN: Post treatment pathological Tumor and Nodal stage
CRC: Colorectal cancer
RC: Rectal cancer
RT: Radiotherapy
CRT: Chemoradiotherapy
TNT: Total neoadjuvant therapy
scRT: Short course radiotherapy
LoG: Laplacian of Gaussian
LBP: local binary pattern
mRMR: Minimum redundancy maximum relevance
LASSO: least absolute shrinkage and selection operator
DL: Deep Learning

## References

[1] Brooke E Sylvester and E. Vakiani, ‘Tumor evolution and intratumor heterogeneity in colorectal carcinoma: insights from comparative genomic profiling of primary tumors and matched metastases’, *Journal of Gastrointestinal Oncology*, vol. 6, no. 6, 2015, 10.3978/j.issn.2078-6891.2015.083. 10.3978/j.issn.2078-6891.2015.083 PMC4671856 26697200

[2] K. Naxerova *et al.*, ‘Hypermutable DNA chronicles the evolution of human colon cancer’, *Proc. Natl. Acad. Sci. U.S.A.*, vol. 111, no. 18, May 2014, 10.1073/pnas.1400179111. 10.1073/pnas.1400179111 PMC4020055 24753616

[3] T. Morikawa *et al.*, ‘Prognostic Significance and Molecular Associations of Tumor Growth Pattern in Colorectal Cancer’, *Ann Surg Oncol*, vol. 19, no. 6, pp. 1944–1953, Jun. 2012, 10.1245/s10434-011-2174-5. 22189472 10.1245/s10434-011-2174-5 PMC3321113

[4] M. Greaves, ‘Evolutionary Determinants of Cancer’, *Cancer Discovery*, vol. 5, no. 8, pp. 806–820, Aug. 2015, 10.1158/2159-8290.CD-15-0439. 10.1158/2159-8290.CD-15-0439 PMC4539576 26193902

[5] S. Ogino *et al.*, ‘Lymphocytic Reaction to Colorectal Cancer Is Associated with Longer Survival, Independent of Lymph Node Count, Microsatellite Instability, and CpG Island Methylator Phenotype’, *Clinical Cancer Research*, vol. 15, no. 20, pp. 6412–6420, Oct. 2009, 10.1158/1078-0432.CCR-09-1438. 10.1158/1078-0432.CCR-09-1438 PMC2771425 19825961

[6] B. Mlecnik *et al.*, ‘Histopathologic-Based Prognostic Factors of Colorectal Cancers Are Associated With the State of the Local Immune Reaction’, *JCO*, vol. 29, no. 6, pp. 610–618, Feb. 2011, 10.1200/JCO.2010.30.5425. 10.1200/JCO.2010.30.5425 21245428

[7] X. Li *et al.*, ‘Prognostic and predictive value of the macroscopic growth pattern in patients undergoing curative resection of colorectal cancer: a single-institution retrospective cohort study of 4,080 Chinese patients’, *CMAR*, vol. Volume 10, pp. 1875–1887, Jul. 2018, 10.2147/CMAR.S165279. 10.2147/CMAR.S165279 PMC6037271 30013394

[8] M. J. M. Van Der Valk *et al.*, ‘Long-term outcomes of clinical complete responders after neoadjuvant treatment for rectal cancer in the International Watch & Wait Database (IWWD): an international multicentre registry study’, *The Lancet*, vol. 391, no. 10139, pp. 2537–2545, Jun. 2018, 10.1016/S0140-6736(18)31078-X. 10.1016/S0140-6736(18)31078-X 29976470

[9] V. S. Jayaprakasam, J. Alvarez, D. M. Omer, M. J. Gollub, J. J. Smith, and I. Petkovska, ‘Watch-and-Wait Approach to Rectal Cancer: The Role of Imaging’, *Radiology*, vol. 307, no. 1, p. e221529, Apr. 2023, 10.1148/radiol.221529. 10.1148/radiol.221529 PMC10068893 36880951

[10] T. J. George, C. J. Allegra, and G. Yothers, ‘Neoadjuvant Rectal (NAR) Score: a New Surrogate Endpoint in Rectal Cancer Clinical Trials’, *Curr Colorectal Cancer Rep*, vol. 11, no. 5, pp. 275–280, Oct. 2015, 10.1007/s11888-015-0285-2. 10.1007/s11888-015-0285-2 PMC4550644 26321890

[11] I. Imam, K. Hammarström, and B. Glimelius, ‘Determinants of Pre-Surgical Treatment in Primary Rectal Cancer: A Population-Based Study’, *Cancers*, vol. 15, no. 4, p. 1154, Feb. 2023, 10.3390/cancers15041154. 10.3390/cancers15041154 PMC9954598 36831497

[12] B. Glimelius, ‘Recent advances in rectal cancer treatment – are we on the right track?’, *ujms*, vol. 129, p. e10537, Feb. 2024, 10.48101/ujms.v129.10537. 10.48101/ujms.v129.10537 PMC10916366 38449909

[13] R. G. H. Beets-Tan *et al.*, ‘Magnetic resonance imaging for clinical management of rectal cancer: Updated recommendations from the 2016 European Society of Gastrointestinal and Abdominal Radiology (ESGAR) consensus meeting’, *Eur Radiol*, vol. 28, no. 4, pp. 1465–1475, Apr. 2018, 10.1007/s00330-017-5026-2. 29043428 10.1007/s00330-017-5026-2 PMC5834554

[14] G. Brown *et al.*, ‘Morphologic Predictors of Lymph Node Status in Rectal Cancer with Use of High-Spatial-Resolution MR Imaging with Histopathologic Comparison’, *Radiology*, vol. 227, no. 2, pp. 371–377, May 2003, 10.1148/radiol.2272011747. 10.1148/radiol.2272011747 12732695

[15] J. E. Van Timmeren, D. Cester, S. Tanadini-Lang, H. Alkadhi, and B. Baessler, ‘Radiomics in medical imaging—“how-to” guide and critical reflection’, *Insights Imaging*, vol. 11, no. 1, p. 91, Dec. 2020, 10.1186/s13244-020-00887-2. 32785796 10.1186/s13244-020-00887-2 PMC7423816

[16] J. Shin *et al.*, ‘MRI Radiomics Model Predicts Pathologic Complete Response of Rectal Cancer Following Chemoradiotherapy’, *Radiology*, vol. 303, no. 2, pp. 351–358, May 2022, 10.1148/radiol.211986. 10.1148/radiol.211986 35133200

[17] H. Shaish *et al.*, ‘Radiomics of MRI for pretreatment prediction of pathologic complete response, tumor regression grade, and neoadjuvant rectal score in patients with locally advanced rectal cancer undergoing neoadjuvant chemoradiation: an international multicenter study’, *Eur Radiol*, vol. 30, no. 11, pp. 6263–6273, Nov. 2020, 10.1007/s00330-020-06968-6. 10.1007/s00330-020-06968-6 32500192

[18] Y. Cui *et al.*, ‘Radiomics analysis of multiparametric MRI for prediction of pathological complete response to neoadjuvant chemoradiotherapy in locally advanced rectal cancer’, *Eur Radiol*, vol. 29, no. 3, pp. 1211–1220, Mar. 2019, 10.1007/s00330-018-5683-9. 10.1007/s00330-018-5683-9 30128616

[19] Z. Liu *et al.*, ‘Radiomics Analysis for Evaluation of Pathological Complete Response to Neoadjuvant Chemoradiotherapy in Locally Advanced Rectal Cancer’, *Clinical Cancer Research*, vol. 23, no. 23, pp. 7253–7262, Nov. 2017, 10.1158/1078-0432.CCR-17-1038. 10.1158/1078-0432.CCR-17-1038 28939744

[20] W. C. Chong *et al.*, ‘A comprehensive evaluation of MR-radiomics role in NAR score prediction in locally advanced rectal cancer’, *The Royal College of Radiologists Open*, vol. 1, p. 100004, 2023, 10.1016/j.rcro.2023.100004.

[21] Z. Shu, D. Mao, Q. Song, Y. Xu, P. Pang, and Y. Zhang, ‘Multiparameter MRI-based radiomics for preoperative prediction of extramural venous invasion in rectal cancer’, *Eur Radiol*, vol. 32, no. 2, pp. 1002–1013, Feb. 2022, 10.1007/s00330-021-08242-9. 34482429 10.1007/s00330-021-08242-9

[22] H. Yuan, Y. Peng, X. Xu, S. Tu, Y. Wei, and Y. Ma, ‘A Tumoral and Peritumoral CT-Based Radiomics and Machine Learning Approach to Predict the Microsatellite Instability of Rectal Carcinoma’, *CMAR*, vol. Volume 14, pp. 2409–2418, Aug. 2022, 10.2147/CMAR.S377138. 10.2147/CMAR.S377138 PMC9375564 35971393

[23] N. Braman *et al.*, ‘Association of Peritumoral Radiomics With Tumor Biology and Pathologic Response to Preoperative Targeted Therapy for *HER2 (ERBB2)*–Positive Breast Cancer’, *JAMA Netw Open*, vol. 2, no. 4, p. e192561, Apr. 2019, 10.1001/jamanetworkopen.2019.2561. 10.1001/jamanetworkopen.2019.2561 PMC6481453 31002322

[24] N. Horvat *et al.*, ‘MR Imaging of Rectal Cancer: Radiomics Analysis to Assess Treatment Response after Neoadjuvant Therapy’, *Radiology*, vol. 287, no. 3, pp. 833–843, Jun. 2018, 10.1148/radiol.2018172300. 10.1148/radiol.2018172300 PMC5978457 29514017

[25] K. Hammarström, I. Imam, N. Korsavidou Hult, J. Ekström, T. Sjöblom, and B. Glimelius, ‘Determining the use of preoperative (chemo)radiotherapy in primary rectal cancer according to national and international guidelines’, *Radiotherapy and Oncology*, vol. 136, pp. 106–112, Jul. 2019, 10.1016/j.radonc.2019.03.036. 10.1016/j.radonc.2019.03.036 31015111

[26] R. R. Bahadoer *et al.*, ‘Short-course radiotherapy followed by chemotherapy before total mesorectal excision (TME) versus preoperative chemoradiotherapy, TME, and optional adjuvant chemotherapy in locally advanced rectal cancer (RAPIDO): a randomised, open-label, phase 3 trial’, *The Lancet Oncology*, vol. 22, no. 1, pp. 29–42, Jan. 2021, 10.1016/S1470-2045(20)30555-6. 10.1016/S1470-2045(20)30555-6 33301740

[27] B. Glimelius *et al.*, ‘Total neoadjuvant treatment using short-course radiotherapy and four CAPOX cycles in locally advanced rectal cancer with high-risk criteria for recurrence: a Swedish nationwide cohort study (LARCT-US)’, *eClinicalMedicine*, vol. 75, Sep. 2024, 10.1016/j.eclinm.2024.102771. 10.1016/j.eclinm.2024.102771 PMC11577565 39568777

[28] ‘nationellt-vardprogram-tjock-andtarmscancer.pdf’. Accessed: Sep. 05, 2024. [Online]. Available: https://kunskapsbanken.cancercentrum.se/globalassets/cancerdiagnoser/tjock--och-andtarm-anal/vardprogram/nationellt-vardprogram-tjock-andtarmscancer.pdf

[29] A. K. Anagnostopoulos *et al.*, ‘Radiomics/Radiogenomics in Lung Cancer: Basic Principles and Initial Clinical Results’, *Cancers*, vol. 14, no. 7, p. 1657, Mar. 2022, 10.3390/cancers14071657. 10.3390/cancers14071657 PMC8997041 35406429

[30] Y. Zhu *et al.*, ‘Different radiomics annotation methods comparison in rectal cancer characterisation and prognosis prediction: a two-centre study’, *Insights into Imaging*, vol. 15, no. 1, p. 211, Aug. 2024, 10.1186/s13244-024-01795-5. 10.1186/s13244-024-01795-5 PMC11347551 39186173

[31] A. Delli Pizzi *et al.*, ‘MRI-based clinical-radiomics model predicts tumor response before treatment in locally advanced rectal cancer’, Sci Rep, vol. 11, no. 1, p. 5379, Mar. 2021, 10.1038/s41598-021-84816-3. 33686147 10.1038/s41598-021-84816-3 PMC7940398

[32] V. S. Jayaprakasam *et al.*, ‘MRI radiomics features of mesorectal fat can predict response to neoadjuvant chemoradiation therapy and tumor recurrence in patients with locally advanced rectal cancer’, *Eur Radiol*, vol. 32, no. 2, pp. 971–980, Feb. 2022, 10.1007/s00330-021-08144-w. 10.1007/s00330-021-08144-w PMC9018044 34327580

[33] P. D. Mc Entee *et al.*, ‘Extramural venous invasion (EMVI) in colorectal cancer is associated with increased cancer recurrence and cancer-related death’, European Journal of Surgical Oncology, vol. 48, no. 7, pp. 1638–1642, Jul. 2022, 10.1016/j.ejso.2022.02.013. 35249791 10.1016/j.ejso.2022.02.013

[34] U. Karjol, P. Jonnada, A. Chandranath, and S. Cherukuru, ‘Lymph Node Ratio as a Prognostic Marker in Rectal Cancer Survival: A Systematic Review and Meta-Analysis’, *Cureus*, May 2020, 10.7759/cureus.8047. 10.7759/cureus.8047 PMC7216312 32399378

[35] N. Papanikolaou, C. Matos, and D. M. Koh, ‘How to develop a meaningful radiomic signature for clinical use in oncologic patients’, Cancer Imaging, vol. 20, no. 1, p. 33, Dec. 2020, 10.1186/s40644-020-00311-4. 32357923 10.1186/s40644-020-00311-4 PMC7195800

[36] B. Kocak *et al.*, ‘METhodological RadiomICs Score (METRICS): a quality scoring tool for radiomics research endorsed by EuSoMII’, *Insights Imaging*, vol. 15, no. 1, p. 8, Jan. 2024, 10.1186/s13244-023-01572-w. 10.1186/s13244-023-01572-w PMC10792137 38228979

[37] F. Maleki, K. Ovens, R. Gupta, C. Reinhold, A. Spatz, and R. Forghani, ‘Generalizability of Machine Learning Models: Quantitative Evaluation of Three Methodological Pitfalls’, *Radiology: Artificial Intelligence*, vol. 5, no. 1, p. e220028, Jan. 2023, 10.1148/ryai.220028. 10.1148/ryai.220028 PMC9885377 36721408

