# Supplementary material for: Inclusion of tumor periphery in radiomics analysis of magnetic resonance images does not improve predictions of preoperative therapy response in patients with rectal cancer
Source: Abdom Radiol (NY). 2025 Feb 5;51(3):1116–28. doi: 10.1007/s00261-025-04815-0 (PMC12971816; doi:10.1007/s00261-025-04815-0)
Supplement: Supplementary file 1 — Supplementary Material 1 [file 261_2025_4815_MOESM1_ESM.docx]

Supplement material

Inclusion of tumor periphery in radiomics analysis of magnetic resonance images does not improve predictions of preoperative therapy response in patients with rectal cancer

Nafsika Korsavidou-Hult^a^, Sambit Tarai^a^, Klara Hammarström^b^, Joel Kullberg^a,d^, Elin Lundström^a^, Tomas Bjerner^c,a^,  Bengt  Glimelius^b^ , Håkan Ahlström^a,d^

^a^Radiology, Department of Surgical Sciences, Uppsala University, Sweden
^b^Department of Immunology, Genetics and Pathology, Uppsala University, Sweden

^c^Dept. of Health, Medicine and Caring Sciences (HMV), Division of Diagnostics and Specialist Medicine (DISP), Linköping University, Sweden

^d^Antaros Medical AB, Mölndal, Sweden

Radiomics features

**Top features selected by mRMR**

‘log-sigma-5-mm-3D_gldm_GrayLevelVariance’,

‘wavelet-LH_firstorder_Skewness’,

‘square_glrlm_GrayLevelVariance’,

‘log-sigma-1-mm-3D_gldm_DependenceVariance’,

‘wavelet-HL_firstorder_Skewness’,

‘lbp-2D_glcm_ClusterProminence’,

‘log-sigma-3-mm-3D_glcm_SumAverage’,

‘lbp-2D_glcm_ClusterTendency’,

‘wavelet-LH_firstorder_Uniformity’,

‘lbp-2D_gldm_HighGrayLevelEmphasis’,

‘log-sigma-5-mm-3D_gldm_LargeDependenceLowGrayLevelEmphasis’,

‘logarithm_glrlm_RunEntropy’,

‘lbp-2D_gldm_GrayLevelVariance’,

‘lbp-2D_firstorder_RobustMeanAbsoluteDeviation’,

‘square_glcm_ClusterShade’,

‘lbp-2D_firstorder_Uniformity’,

‘original_glrlm_RunVariance’,

‘log-sigma-5-mm-3D_firstorder_InterquartileRange’,

‘lbp-2D_glcm_ClusterShade’,

‘lbp-2D_firstorder_Range’

**Top features selected by LASSO**

'wavelet-HL_glcm_Idmn',

'log-sigma-1-mm-3D_glcm_Idn',

'wavelet-HH_glcm_Imc1',

'wavelet-LH_ngtdm_Contrast',

'wavelet-LH_glcm_MaximumProbability',

'exponential_glszm_SmallAreaEmphasis',

'original_glcm_InverseVariance',

'wavelet-HH_glszm_SmallAreaEmphasis',

'log-sigma-3-mm-3D_glszm_LowGrayLevelZoneEmphasis', 'exponential_glszm_SmallAreaLowGrayLevelEmphasis',

'log-sigma-5-mm-3D_ngtdm_Strength',

'log-sigma-5-mm-3D_glcm_ClusterShade',

'log-sigma-1-mm-3D_glcm_MaximumProbability',

'square_firstorder_Uniformity',

'log-sigma-1-mm-3D_glcm_InverseVariance',

'wavelet-HL_glrlm_RunEntropy',

'log-sigma-1-mm-3D_glcm_Correlation',

‘T_stage’

**Top features selected by Logistic Regression**

'log-sigma-5-mm-3D_glcm_ClusterProminence',

'lbp-2D_firstorder_MeanAbsoluteDeviation',

'log-sigma-3-mm-3D_glszm_LowGrayLevelZoneEmphasis',

'log-sigma-1-mm-3D_firstorder_Skewness',

'wavelet-LH_ngtdm_Complexity',

'log-sigma-5-mm-3D_glrlm_ShortRunHighGrayLevelEmphasis', '

log-sigma-3-mm-3D_gldm_LargeDependenceLowGrayLevelEmphasis',

'logarithm_firstorder_TotalEnergy',

'wavelet-LH_firstorder_Maximum',

'log-sigma-1-mm-3D_glcm_InverseVariance',

'wavelet-HL_firstorder_Skewness',

'lbp-2D_glcm_JointEntropy',

'squareroot_gldm_LargeDependenceHighGrayLevelEmphasis',

'log-sigma-3-mm-3D_ngtdm_Busyness',

'log-sigma-3-mm-3D_glszm_SmallAreaLowGrayLevelEmphasis',

'log-sigma-5-mm-3D_glcm_ClusterShade',

'square_gldm_DependenceVariance',

'wavelet-HL_glcm_ClusterShade'
