# Supplementary material for: Inclusion of tumor periphery in radiomics analysis of magnetic resonance images does not improve predictions of preoperative therapy response in patients with rectal cancer
Source: Abdom Radiol (NY). 2025 Feb 5;51(3):1116–28. doi: 10.1007/s00261-025-04815-0 (PMC12971816; doi:10.1007/s00261-025-04815-0)
Supplement: Supplementary file 2 — Supplementary Material 2 [file 261_2025_4815_MOESM2_ESM.docx]

Supplement material

Inclusion of tumor periphery in radiomics analysis of magnetic resonance images does not improve predictions of preoperative therapy response in patients with rectal cancer

Nafsika Korsavidou-Hult^a^, Sambit Tarai^a^, Klara Hammarström^b^, Joel Kullberg^a,d^, Elin Lundström^a^, Tomas Bjerner^c,a^,  Bengt  Glimelius^b^ , Håkan Ahlström^a,d^

^a^Radiology, Department of Surgical Sciences, Uppsala University, Sweden
^b^Department of Immunology, Genetics and Pathology, Uppsala University, Sweden

^c^Dept. of Health, Medicine and Caring Sciences (HMV), Division of Diagnostics and Specialist Medicine (DISP), Linköping University, Sweden

^d^Antaros Medical AB, Mölndal, Sweden

Supplementary material A.

Radiomics features

**Top features selected by mRMR**

‘log-sigma-5-mm-3D_gldm_GrayLevelVariance’,

‘wavelet-LH_firstorder_Skewness’,

‘square_glrlm_GrayLevelVariance’,

‘log-sigma-1-mm-3D_gldm_DependenceVariance’,

‘wavelet-HL_firstorder_Skewness’,

‘lbp-2D_glcm_ClusterProminence’,

‘log-sigma-3-mm-3D_glcm_SumAverage’,

‘lbp-2D_glcm_ClusterTendency’,

‘wavelet-LH_firstorder_Uniformity’,

‘lbp-2D_gldm_HighGrayLevelEmphasis’,

‘log-sigma-5-mm-3D_gldm_LargeDependenceLowGrayLevelEmphasis’,

‘logarithm_glrlm_RunEntropy’,

‘lbp-2D_gldm_GrayLevelVariance’,

‘lbp-2D_firstorder_RobustMeanAbsoluteDeviation’,

‘square_glcm_ClusterShade’,

‘lbp-2D_firstorder_Uniformity’,

‘original_glrlm_RunVariance’,

‘log-sigma-5-mm-3D_firstorder_InterquartileRange’,

‘lbp-2D_glcm_ClusterShade’,

‘lbp-2D_firstorder_Range’

**Top features selected by LASSO**

'wavelet-HL_glcm_Idmn',

'log-sigma-1-mm-3D_glcm_Idn',

'wavelet-HH_glcm_Imc1',

'wavelet-LH_ngtdm_Contrast',

'wavelet-LH_glcm_MaximumProbability',

'exponential_glszm_SmallAreaEmphasis',

'original_glcm_InverseVariance',

'wavelet-HH_glszm_SmallAreaEmphasis',

'log-sigma-3-mm-3D_glszm_LowGrayLevelZoneEmphasis', 'exponential_glszm_SmallAreaLowGrayLevelEmphasis',

'log-sigma-5-mm-3D_ngtdm_Strength',

'log-sigma-5-mm-3D_glcm_ClusterShade',

'log-sigma-1-mm-3D_glcm_MaximumProbability',

'square_firstorder_Uniformity',

'log-sigma-1-mm-3D_glcm_InverseVariance',

'wavelet-HL_glrlm_RunEntropy',

'log-sigma-1-mm-3D_glcm_Correlation',

‘T_stage’

**Top features selected by Logistic Regression**

'log-sigma-5-mm-3D_glcm_ClusterProminence',

'lbp-2D_firstorder_MeanAbsoluteDeviation',

'log-sigma-3-mm-3D_glszm_LowGrayLevelZoneEmphasis',

'log-sigma-1-mm-3D_firstorder_Skewness',

'wavelet-LH_ngtdm_Complexity',

'log-sigma-5-mm-3D_glrlm_ShortRunHighGrayLevelEmphasis', '

log-sigma-3-mm-3D_gldm_LargeDependenceLowGrayLevelEmphasis',

'logarithm_firstorder_TotalEnergy',

'wavelet-LH_firstorder_Maximum',

'log-sigma-1-mm-3D_glcm_InverseVariance',

'wavelet-HL_firstorder_Skewness',

'lbp-2D_glcm_JointEntropy',

'squareroot_gldm_LargeDependenceHighGrayLevelEmphasis',

'log-sigma-3-mm-3D_ngtdm_Busyness',

'log-sigma-3-mm-3D_glszm_SmallAreaLowGrayLevelEmphasis',

'log-sigma-5-mm-3D_glcm_ClusterShade',

'square_gldm_DependenceVariance',

'wavelet-HL_glcm_ClusterShade'

Supplementary material B.

Segmentation examples

B1

B2

B3

B4

B5

Segmentation examples for 5 different patients. First row from left to right: T2 weighted image without any annotation. T2 weighted image with the wROI annotation. Second row from left to right: Diffusion weighted image (DWI) and T2 weighted image with cROI annotation.

*wROI : whole tumor. The tumor was delineated to its outmost margin, including also the tumor extensions into the mesorectum. cROI : central part of the tumor.* The tumor was delineated on T2w images including the parts with high signal voxels from the spatially registered DWI (b-value 800 s/mm^2^) and excluding approximately 2-4 mm tumor rim.

Supplementary material C.

Fig 5 Good and Bad performance of the cROIs when predicting different outcomes.

Model performances in the different outcomes on different patients. The images represent axial T2 weighted images with the cROI (central tumor region of interest). The cROI segmentations accurately predict the achieved outcome t.ex pCR (yes) and non-pCR(no) as the chosen binary classification of all the three outcomes.
